# Regional employment and individual worklessness during the Great Recession and the health of the working-age population: Cross-national analysis of 16 European countries

**DOI:** 10.1016/j.socscimed.2019.112377

**Published:** 2020-12

**Authors:** Claire L. Niedzwiedz, Katie H. Thomson, Clare Bambra, Jamie R. Pearce

**Affiliations:** a Institute of Health & Wellbeing, University of Glasgow, Glasgow, UK; b Centre for Research on Environment, Society and Health, University of Edinburgh, Edinburgh, UK; c Institute of Health and Society, Faculty of Medical Sciences, Newcastle University, Newcastle, UK

**Keywords:** Health inequality, Employment, Geography, Cross-national, Worklessness, Recession

## Abstract

Studies from single countries suggest that local labour market conditions, including rates of employment, tend to be associated with the health of the populations residing in those areas, even after adjustment for individual characteristics including employment status. The aim of this study is to strengthen the cross-national evidence base on the influence of regional employment levels and individual worklessness on health during the period of the Great Recession. We investigate whether higher regional employment levels are associated with better health over and above individual level employment. Individual level data (N = 23,078 aged 15–64 years) were taken from 16 countries (Austria, Belgium, Czech Republic, Denmark, Finland, France, Germany, Hungary, Ireland, Netherlands, Norway, Poland, Portugal, Spain, Sweden and United Kingdom) participating in the 2014 European Social Survey. Regional employment rates were extracted from Eurostat, corresponding with the start (2008) and end (2013) of the Great Recession. Health outcomes included self-reported heart or circulation problems, high blood pressure, diabetes, self-rated health, depression, obesity and allergies (as a falsification test). We calculated multilevel Poisson regression models, which included individuals nested within regions, controlling for potential confounding variables and country fixed effects. After adjustment for individual level socio-demographic factors, higher average regional employment rates (from 2008 to 2013) were associated with better health outcomes. Individual level worklessness was associated with worsened health outcomes, most strongly with poor self-rated health. In models including both individual worklessness and the average regional employment rate, regional employment remained associated with heart and circulation problems, depression and obesity. There was evidence of an interaction between individual worklessness and regional employment for poor self-rated health and depression. The findings suggest that across 16 European countries, for some key outcomes, higher levels of employment in the regional labour market may be beneficial for the health of the local population.

## Highlights

• Few cross-national studies have examined regional employment and health.
• High regional employment is related to reduced risk of poor health.
• Regional employment moderates the impact of individual worklessness on some outcomes.

## 1 Introduction

Since the onset of the global financial crisis in 2008 and the subsequent recession experienced in many countries, there has been renewed interest in the role of unemployment, job insecurity and worklessness in influencing population health (Bambra, 2011). This study aims to strengthen the cross-national evidence base on the influence of individual worklessness and regional employment levels (as a measure of the local labour market) on health during the Great Recession.

### 1.1 Individual level worklessness

Worklessness - being outside the labour market due to unemployment (out of work but actively looking for a job); lone parenthood; and long-term disability (Gabbay et al., 2011), is associated with poorer health and social exclusion (Bambra, 2011). Findings from many different settings over the past few decades have demonstrated the deleterious impact of unemployment on a range of health outcomes including overall mortality (Roelfs et al., 2011), suicide (Platt, 1984), cardiovascular disease (Dupre et al., 2012), common mental health disorders (Fone et al., 2007), psychological distress (Thomas et al., 2007), adverse health behaviours such as alcohol and tobacco consumption (Montgomery et al., 1998; Popovici and French, 2013), poor self-rated health (Bambra and Eikemo, 2008) and elevated inflammatory biomarkers (Hughes et al., 2017). Links between unemployment and poor health have conventionally been explained through two inter-related concepts: the psychosocial effects of unemployment (e.g. stigma, isolation and loss of self-worth) and the material consequences of unemployment (e.g. wage loss and resulting changes in access to essential goods and services) (Bambra, 2011).

Other aspects of worklessness are also linked to poorer health and employment outcomes. For example, lone mothers are twice as likely as coupled mothers to describe their health as ‘not good’ and across Europe, a range of adverse health outcomes are experienced disproportionately by lone parents, including psychiatric disorders; attempted suicide; and alcohol and drugs-related disease (Gibson et al., 2012). Mechanisms linking lone parenthood to poor health also include poverty, often due to non-employment, lack of support, and stigma (Benzeval, 1998). Further, having a long-term health condition or disability is a significant risk factor for being out of the labour market. People who develop chronic health problems whilst in employment are twice as likely to become workless within a four year period as those who remain healthy, and women and men in poor health are 60% and 40% less likely to enter paid employment than those in good health (Schuring et al., 2009). In combination with other labour market disadvantages such as low educational level, poor health further increases the risk of worklessness and there are substantial regional inequalities in health-related worklessness (Bambra and Popham, 2010).

### 1.2 Local and regional labour market context

More recently, research has shown that local labour market conditions, including rates of employment and unemployment, tend to be associated with the physical and mental health outcomes of the populations residing in those areas, even after adjustment for individual characteristics including employment status (Cummins et al., 2005; Flint et al., 2013). Understanding the role of local labour market conditions in influencing health is important because the health impacts of economic downturns in some places may be larger and more prolonged than in other places, which has significant policy implications. However, to date, the international evidence base examining the relationships between local labour market conditions and health is slight, and to our knowledge there are no international comparative studies investigating the individual and contextual influence of employment on individual level health status.

Local labour market conditions are likely to influence population health through a number of interrelated pathways including: heightened job insecurity; weakening community cohesion; increasing place-based stigma; health selective migration; stifling regional income levels; raising workplace stresses; accentuating local problems related to poverty such as crime, unhealthy behaviours (e.g. tobacco and alcohol use) and illicit drug use; and detrimentally affecting the local economy by restricting the availability of community resources such as shops (Riva and Curtis, 2012).

The evidence base linking contextual employment to health is mixed and it is clear that, for some health outcomes at least, the relationships between local employment opportunities and health are complex. Studies within the same country have found conflicting results; in Sweden two studies found that after adjusting for individual employment status, local unemployment levels were associated with adverse outcomes including coronary heart disease (Sundquist et al., 2006) and smoking (Öhlander et al., 2006), whereas another study of psychological distress did not find an association (Strandh et al., 2011). However, psychiatric disorders, suicide and parasuicide have been found to be less prevalent for unemployed people in areas of high unemployment compared to low unemployment areas (Jackson and Warr, 1987; Platt and Kreitman, 1990; Platt et al., 1992; Powdthavee, 2007). One interpretation of this is that in areas where unemployment levels are high, unemployment is less stigmatised, and the impact of an individual's own unemployment is diminished (Flint et al., 2013). However, in a study exploring the impact of unemployment on subjective wellbeing in Germany and Switzerland, high regional unemployment levels did not act as a buffer for those who became unemployed (Oesch and Lipps, 2013), going against this hypothesis.

Importantly, recent work has emphasised that the long-term employment trajectories in local labour market conditions may be important for health. A longitudinal study of trends in local labour market conditions between 1981 and 2008 in England found that the risk of mortality or morbidity was greater in places where employment rates were persistently low or declining (Riva and Curtis, 2012). Similarly, work using a Swedish cohort found that high levels of local unemployment had a detrimental impact on functional somatic symptoms (bodily complaints such as headaches, musculoskeletal pain, abdominal pain and dizziness) and this association was strongest in adulthood at age 30 (Brydsten et al., 2017). There is also evidence that the influence of local labour market conditions on health may vary between different sociodemographic groups. Local labour market conditions are likely to be particularly important for those who are economically inactive or job insecure, as well as those who are less mobile or tend to face greater work-based discrimination (e.g. women, ethnic minority groups and those with low levels of relevant skills or education). Considering the potential impact of local labour market conditions on health is vital during times of economic downturn as regions may have differing levels of resilience to economic shocks depending on their baseline level of employment and the type of employment which dominates (e.g. manufacturing and public sector), as well as the differential response to the recession across regions, including the implementation of austerity measures characterised by public expenditure cuts to reduce government debt (Blažek and Netrdová, 2012; Davies, 2011).

### 1.3 The Great Recession and austerity

National economic wealth (i.e. Gross Domestic Product) has long been considered as a major global determinant of population health, with the vast differences in mortality between developed and developing countries accounted for in terms of differences in economic growth. Changes in the economy therefore potentially have important implications for population health and inequalities in health. Economic recessions are characterised by instability (in terms of inflation and interest rates) and sudden reductions in production and consumption with corresponding increases in unemployment. The economic downturn which started in late 2007 is popularly referred to as the ‘Great Recession’ as it has been longer, wider and deeper than any previous economic downturns including the ‘Great Depression’ of the 1930s (Gamble, 2009). For example, it was characterised by unemployment rates of around 8.5% in the UK and the USA, 10% in France and more than 20% in Spain.

The short term overall population health effects of recessions are rather mixed. The majority of international studies conclude that there are declines in all-cause mortality, deaths from cardiovascular disease and motor vehicle accidents, as well as decreases in hazardous health behaviours during economic downturns, whilst deaths from suicides, rates of mental ill health and chronic illnesses increase (Bambra et al., 2016). Following the 2007/8 crisis, a worldwide an excess of 4884 suicides was observed in 2009 (Corcoran et al., 2015) and over the next 3 years (2008–2010) an excess of 4750 suicides occurred in the USA, 1000 suicides in England, and 680 suicides in Spain (Lopez Bernal et al., 2013; Reeves et al., 2012). There is also evidence of other increases in poor mental health and wellbeing after the ‘Great Recession’ including self-harm and psychiatric morbidity (Barnes et al., 2017; Katikireddi et al., 2012).

However, studies have found that there are important variations in the effects of recessions and economic downturns on population health – depending on policy responses. In a wide ranging and well publicised analysis of the health effects of austerity, Stuckler and Basu (2013) concluded that the overall effects of recessions on the health of different nations vary significantly by political and policy context, with those countries (such as Iceland or the USA) who responded to the financial crisis of 2007/8 with an economic stimulus, faring much better - particularly in terms of mental health and suicides - than those countries (e.g. Spain, Greece or UK) who chose to pursue austerity. Similarly, Karanikolos et al. (2013) found that across Europe, weak social protection systems increased the health and social crisis in Europe. Whilst, previously, Hopkins (2006) found that in Thailand and Indonesia where social welfare spending was decreased during the Asian recession of the late 1990s, mortality rates increased. However, in Malaysia where no cut backs occurred, mortality rates were unchanged (Hopkins, 2006). Similarly, a study of 26 European countries concluded that greater spending on social welfare could considerably reduce suicide rates during periods of economic downturn (Stuckler et al., 2009). In the UK, there is evidence that the pressures that austerity has placed on key social and health care services resulted in up to 10,000 additional deaths in 2018 compared to previous years (Hiam and Dorling, 2018). However, there is little evidence on the differential regional impact of the recession. In the UK, areas with higher unemployment rates experienced greater increases in suicide (Hawton et al., 2016) and studies have demonstrated the mixed effect the recession has had on regions across Europe depending on political decisions and existing institutional frameworks (Davies, 2011).

### 1.4 Aims and objectives

The aim of this study is to strengthen the evidence base on the influence of regional employment levels (as a measure of the local labour market) and individual worklessness on health by examining these relationships across 16 European countries during the Great Recession. Specifically, we have four key objectives:

1. Examine the influence of regional employment levels and individual worklessness on the health of the working-age population across Europe.
2. Examine whether regional employment levels at different time points, the employment rate across the whole recessionary period, or the change in employment rate during a period of recession and austerity, are associatied with health.
3. Investigate whether regional employment levels are associated with health over and above individual worklessness.
4. Examine whether there is an interaction between individual level worklessness and the regional employment rate.

## 2 Methods

### 2.1 Data

Individual level data were taken from Round 7 (edition 2.1) of the European Social Survey (ESS) which was collected during 2014/15 (European Social Survey, 2014). Data are openly available and can be accessed by visiting https://www.europeansocialsurvey.org. We included data from 16 European countries (Austria, Belgium, Czech Republic, Denmark, Finland, France, Germany, Hungary, Ireland, Netherlands, Norway, Poland, Portugal, Spain, Sweden and United Kingdom). We excluded Estonia, Israel, Switzerland and Lithuania due to the lack of available and comparable regional level data. The regional level data were extracted from Eurostat (https://ec.europa.eu/eurostat/) during 2016. All regional data were classified at the second Nomenclature of Territorial Units for Statistics (NUTS-2) level (with a typical population of 800,000 to 3 million) apart from Germany and United Kingdom, as for these countries the ESS data were only available at the NUTS-1 level (population 3–7 million). The NUTS is a hierarchical system for dividing up the economic territory of the European Union based on population, country administrative divisions or geographical units (Thomson et al., 2017). The regional data were matched with the individual level ESS data using the corresponding NUTS code. We included people aged 15–64 years (N = 23,078).

### 2.2 Outcomes

We included a range of health outcomes present in Round 7 of the ESS, these included: self-reported heart or circulation problems, high blood pressure, diabetes, self-rated health, depression and obesity. The regional distribution for these outcomes has been described previously (Thomson et al., 2017). As a falsification test we also tested an outcome (allergies) that we did not expect to be associated with individual or regional level employment. The first three outcomes (heart or circulation problems, high blood pressure and diabetes) and the allergy falsification test variable were binary variables, whereby participants were coded yes if they reported having the condition over the past 12 months and no if they did not. Poor self-rated health was defined as those reporting their health as bad or very bad, as opposed to very good, good or fair. Depressive symptoms were measured via the Center for Epidemiologic Depression Scale (CESD-8) (Radloff, 1977), which included eight questions relating to how often the participant felt a range of feelings, such as sadness, loneliness, and happiness, over the past week. Positively worded questions were reverse coded and a scale was derived from the sum of the eight items, which ranged from 0 to 24. Potential cases of depression were coded as those scoring a value of 10 or more (Thomson et al., 2017). Obesity was defined as having a Body Mass Index of 30 or more, which was derived from self-reported height and weight.

### 2.3 Exposures

Individuals were asked about their main activity in the past seven days: in paid work, in education, unemployed and actively looking for a job, unemployed and not actively looking for a job, permanently sick or disabled, retired, in community or military service, doing housework, or looking after children or other persons. Individuals who did not report being in paid work were defined as workless and this was used as our main individual level exposure variable. We chose to categorise individuals into those who were workless versus those who were not because we were interested in the combined influence of worklessness and the regional employment level on health, not just unemployment. We chose to keep students in the workless group as we hypothesised that some outcomes (e.g. depression) may be worse among students in areas of low regional employment and this is consistent with a previous study (Fritzell et al., 2012). At the regional level we included the employment rate of those aged 15–64 years during 2008, representing a time period near the beginning of the Great Recession to examine whether this had a lasting association with the health outcomes, and also during 2013, which represents the most recent period preceding the collection of the health outcome data, but also a time at the end of the Great Recession during a period of austerity in some European countries. We also examined the average employment rate between 2008 and 2013 and the difference in the employment rate between 2008 and 2013 to assess whether the change in the employment rate was important over and above the overall rate. We chose to use the employment rate rather than the unemployment rate because employment rates are a more robust indicator of the local labour market and have been used previously in a number of other key studies focused on the influence of the local labour market on health (Curtis et al., 2019; Riva and Curtis, 2012). Unemployment rates alone only capture one aspect of recession and local labour market conditions, whereas employment rates also take into account potential increases in those out of work due to sickness, disability and caring responsibilities. Unemployment rates may also fall when there has been no improvement in the local labour market, as the definition of unemployment only covers those who are out of work and actively seeking employment. When unemployment rates are high, those who are disadvantaged for various reasons (e.g. long-term unemployed, low skills, disability) may become discouraged due to increased competition and cease actively searching for employment, resulting in a fall in the unemployment rate (OECD, 2018). Employment rates are also more likely to be comparable across countries as the definition of the unemployed is more variable between countries (Bambra and Eikemo, 2009).

### 2.4 Confounding variables

We included a number of potential individual level confounding factors: age (years), gender, education level (tertiary versus non-tertiary) and marital status (married/cohabiting versus not married/cohabiting). These individual level variables were considered to be associated with both individual level worklessness and the health outcomes, therefore potentially confounding these relationships. All models also included country dummy variables to control for factors at the country level which may be related to both worklessness and the outcome variables, such as the national employment rate.

### 2.5 Statistical analysis

We firstly examined descriptive statistics, using post-stratification weights, for each variable. We then calculated multilevel random-intercept Poisson regression models for the binary outcome variables, which included individuals nested within NUTS regions. Poisson regression for binary outcome variables enables the calculation of Prevalence Risk Ratios (PRRs), allowing reliable comparison across models and samples, as well as performing well when the outcome is rare (Barros and Hirakata, 2003; Katikireddi et al., 2016). All models included country fixed effects, which control for the variance in the health outcomes attributed to the country level that may be due to factors such as the national unemployment rate, and therefore reducing the likelihood of omitted variable bias. Due to the relatively small number of countries included, it was not feasible to calculate three-level models. We also included robust standard errors (clustered at the regional level) to account for potential violations to heteroskedasticity. We excluded individuals with missing exposure data (N = 296). Models for each outcome variable may therefore contain a different number of individuals as we did not exclude those with missing values for each outcome. All analyses were performed using Stata/MP 15.1.

The following statistical models were calculated for each outcome variable: we first calculated multilevel regression models including only the regional level employment variables in turn, controlling for country fixed effects. This was followed by the calculation of multilevel regression models which included the individual level confounding variables (age, gender, marital status and education level) and country fixed effects. We then added individual worklessness status followed by the regional level employment variable, and then the interaction between the regional employment and individual worklessness. For any statistically significant interactions, we calculated predictive margins and graphed these to aid the interpretation of results. As a sensitivity analysis, we also tested the interaction between the regional employment rate and gender, as well as education level, to investigate whether there were potential moderating effects. For results of interest we broke the workless category down into employed, unemployed, permanently sick or disabled, retired, homemaker or other (e.g. in education, military or community service) to see if any specific group was driving the results, as a sensitivity analysis.

## 3 Results

Across the 16 countries, allergies were the most prevalent health outcome (14.0%) and diabetes (2.9%) the least (Table 1 and Table S1). Heart and circulation problems were most prevalent in Poland (10.5%) and the least in Ireland (2.1%). High blood pressure was highest in Germany (16.4%) and lowest in Ireland (6.0%). Diabetes was most frequently observed in Portugal (5.5%) and the least in Ireland (1.5%). Poor self-rated health was most often observed in Germany (7.7%) and the least in Ireland (1.7%). Depression was most common in Portugal (19.9%) and the least common in Finland (6.9%). Obesity was most prevalent in United Kingdom (18.0%) and the least in Austria (9.1%). Allergies were most common in Portugal (22.6%) and the least common in Hungary and Ireland (5.7%).

**Table 1:** Descriptive statistics (weighted) for each health condition by country.

	Heart/circulation problems		High blood pressure		Diabetes		Poor self-rated health		Depression		Obesity		Allergies	
	% No	% Yes	% No	% Yes	% No	% Yes	% No	% Yes	% No	% Yes	% No	% Yes	% No	% Yes
Austria	95.1	4.9	90.9	9.1	98.4	1.6	96.7	3.3	89.9	10.1	90.9	9.1	90.3	9.7
Belgium	94.1	5.9	87.5	12.5	97.6	2.4	96.1	3.9	89.3	10.7	87.5	12.5	85.4	14.6
Czech Republic	97.1	2.9	90.9	9.1	96.6	3.4	97.1	2.9	82.4	17.6	89.1	10.9	91.5	8.5
Germany	90.2	9.8	83.6	16.4	96.5	3.5	92.3	7.7	86.3	13.7	84.8	15.2	82.0	18.0
Denmark	95.2	4.8	88.4	11.6	95.7	4.3	94.5	5.5	88.6	11.4	87.5	12.5	81.1	18.9
Spain	95.4	4.6	90.7	9.3	97.4	2.6	94.1	5.9	84.2	15.8	86.1	13.9	87.4	12.6
Finland	94.6	5.4	85.5	14.5	95.9	4.1	97.5	2.5	93.1	6.9	82.7	17.3	80.3	19.7
France	94.9	5.1	91.8	8.2	96.8	3.2	94.9	5.1	88.7	11.3	85.4	14.6	85.4	14.6
United Kingdom	95.9	4.1	88.8	11.2	96.7	3.3	94.2	5.8	86.2	13.8	82.0	18.0	87.1	12.9
Hungary	94.1	5.9	86.2	13.8	96.6	3.4	93.4	6.6	81.8	18.2	87.8	12.2	94.3	5.7
Ireland	97.9	2.1	94.0	6.0	98.5	1.5	98.3	1.7	91.5	8.5	89.0	11.0	94.3	5.7
Netherlands	94.2	5.8	88.5	11.5	96.8	3.2	95.9	4.1	91.4	8.6	87.8	12.2	85.4	14.6
Norway	96.2	3.8	90.4	9.6	98.4	1.6	93.5	6.5	91.8	8.2	87.9	12.1	78.1	21.9
Poland	89.5	10.5	87.8	12.2	97.6	2.4	94.7	5.3	85.6	14.4	84.5	15.5	88.6	11.4
Portugal	91.9	8.1	83.8	16.2	94.5	5.5	94.1	5.9	80.1	19.9	85.5	14.5	77.4	22.6
Sweden	96.1	3.9	89.8	10.2	97.8	2.2	96.6	3.4	89.4	10.6	87.3	12.7	80.9	19.1
**Total**	**94.6**	**5.4**	**88.7**	**11.3**	**97.1**	**2.9**	**95.3**	**4.7**	**87.7**	**12.3**	**86.5**	**13.5**	**86.0**	**14.0**

Overall, the prevalence of worklessness was 37.7% and it varied from 31.0% in Austria to 45.9% in Ireland (Table 2). The lowest average regional employment rate in 2008 was observed in Hungary (56.4%), whereas in 2013 it was in Spain (54.7%), but Hungary displayed the lowest average employment rate across the six years. Norway exhibited the highest employment rate at both time periods (78.0% in 2008 but decreasing to 75.4% in 2013). The largest fall in the employment rate between 2008 and 2013 was seen for Spain (9.5%), whereas Germany saw the largest increase (3.6%).

**Table 2:** Descriptive statistics for worklessness (weighted) and regional employment by country.

				2008 employment rate		2013 employment rate		Average employment rate 2008 to 2013		Difference in employment rate 2008 to 2013	
	% Employed	% Workless	N	Mean	SD	Mean	SD	Mean	SD	Mean	SD
Austria	69.0	31.0	1,368	70.9	3.0	71.6	3.2	71.1	3.0	0.6	0.9
Belgium	58.7	41.3	1,400	62.2	5.3	61.8	5.7	61.8	5.4	−0.5	1.1
Czech Republic	65.6	34.4	1,635	66.5	2.8	67.6	3.0	66.1	3.0	1.2	0.8
Germany	62.5	37.5	2,282	70.0	3.1	73.6	2.8	71.9	2.7	3.6	1.2
Denmark	61.7	38.3	1,155	77.8	1.3	72.4	1.4	74.1	1.2	−5.4	0.5
Spain	58.8	41.2	1,483	64.2	5.5	54.7	6.2	58.5	5.8	−9.5	1.6
Finland	61.2	38.8	1,503	71.0	4.1	68.8	3.2	69.1	3.7	−2.3	1.3
France	60.1	39.9	1,438	65.0	3.0	64.2	3.2	64.3	2.9	−0.8	1.8
United Kingdom	65.2	34.8	1,570	71.6	3.1	70.6	2.8	70.2	2.9	−1.0	0.7
Hungary	66.8	33.2	1,242	56.4	5.8	58.0	4.3	56.1	4.9	1.6	1.5
Ireland	54.1	45.9	1,768	67.2	1.2	60.3	1.5	61.0	1.6	−6.9	0.2
Netherlands	60.7	39.3	1,414	77.2	1.7	73.6	1.8	75.1	1.7	−3.6	0.8
Norway	66.5	33.5	1,142	78.0	1.8	75.4	1.7	76.1	1.6	−2.6	1.1
Poland	61.1	38.9	1,272	59.4	3.0	60.1	2.9	59.6	2.8	0.7	1.5
Portugal	59.4	40.6	838	68.1	2.2	60.8	2.3	64.3	2.0	−7.3	0.8
Sweden	68.1	31.9	1,296	74.5	2.1	74.6	2.0	73.6	2.0	0.1	1.0
**Total**	**62.3**	**37.7**	**22,806**	**68.7**	**6.8**	**67.0**	**7.3**	**67.1**	**6.9**	**−1.7**	**3.8**

N=Number of individuals; SD=Standard deviation.

**Fig. 1:**
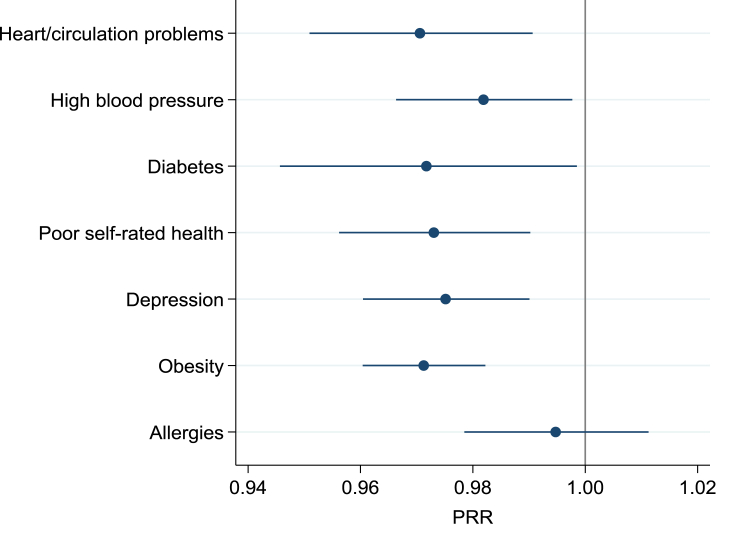
Results from the multilevel regression models examining the average regional employment rate from 2008 to 2013 and health outcomes (95% confidence intervals shown), PRR = prevalence risk ratio.

There was little difference in the strength of the associations when comparing the relationship between the 2008 and 2013 regional employment rates and the health outcomes in the multilevel Poisson regression models (Table 3). The associations observed were in the expected direction, whereby higher regional employment levels were associated with lower risk of poor health and a PRR of below one. The strongest association between the average regional employment rate and the health outcomes was observed for heart and circulation problems where the PRR was 0.971 (95% CI: 0.951 to 0.991) and also obesity with a PRR of 0.971 (95% CI: 0.960 to 0.982). The average regional employment rate was associated with all health outcomes (Fig. 1), apart from allergies (0.995, 95% CI: 0.978 to 1.011). The change in the regional employment rate between 2008 and 2013 was not associated with any of the health outcomes under study, above that of the 2008 regional employment rate. As there was little difference between the different measures of regional employment, we chose to conduct the remaining analysis using the average regional employment rate from 2008 to 2013.

**Table 3:** Results from multilevel regression models investigating regional employment levels and health outcomes.

		Heart or circulation problems	High blood pressure	Diabetes	Poor self-rated health	Depression	Obesity	Allergies
		PRR [95% CI]	PRR [95% CI]	PRR [95% CI]	PRR [95% CI]	PRR [95% CI]	PRR [95% CI]	PRR [95% CI]
Model 1	2008 employment rate	0.973^∗∗^ [0.955,0.991]	0.979^∗∗^ [0.965,0.994]	0.968^∗^ [0.944,0.993]	0.975^∗∗^ [0.959,0.991]	0.975^∗∗^ [0.960,0.990]	0.972^∗∗∗^ [0.960,0.983]	0.996 [0.980,1.012]
Model 2	2013 employment rate	0.973^∗∗^ [0.953,0.993]	0.985 [0.970,1.001]	0.974 [0.947,1.001]	0.975^∗∗^ [0.958,0.993]	0.976^∗∗∗^ [0.962,0.990]	0.972^∗∗∗^ [0.962,0.983]	0.996 [0.980,1.011]
Model 3	Average employment rate (2008–2013)	0.971^∗∗^ [0.951,0.991]	0.982^∗^ [0.966,0.998]	0.972^∗^ [0.946,0.999]	0.973^∗∗^ [0.956,0.990]	0.975^∗∗^ [0.960,0.990]	0.971^∗∗∗^ [0.960,0.982]	0.995 [0.978,1.011]
Model 4	Difference in employment rate (2008–2013)	0.993 [0.943,1.045]	1.046 [1.000,1.095]	1.043 [0.963,1.130]	0.998 [0.948,1.051]	0.994 [0.957,1.032]	0.995 [0.959,1.032]	0.997 [0.964,1.031]
	*N (individuals)*	22433	22433	22433	22793	22483	22169	22433
	*N (regions)*	163	163	163	163	163	163	163

^∗^*p* < 0.05, ^∗∗^*p* < 0.01, ^∗∗∗^*p* < 0.001; CI=Confidence interval; PRR=Prevalence Risk Ratio; All models contain country fixed effects; Model 4 controls for the 2008 employment rate.

We identified gender differences across all health outcomes studied (Table 4, Model 1). Women had higher risk of heart and circulation problems, poor self-rated health, depression and allergies, whereas men had greater risk of high blood pressure, diabetes and obesity. Being married or cohabiting with a partner was associated with lower risk of all health outcomes apart from obesity. Educational inequalities were apparent across all health outcomes apart from allergies, where the higher educated experienced greater risk. The largest educational inequality was seen for poor self-rated health. Adding individual worklessness to the model attenuated the educational inequalities in health (Table 4, Model 2).

**Table 4:** Results from multilevel regression models investigating individual worklessness, regional employment levels and health outcomes.

	Heart or circulation problems	High blood pressure	Diabetes	Poor self-rated health	Depression	Obesity	Allergies
Model 1	PRR [95% CI]	PRR [95% CI]	PRR [95% CI]	PRR [95% CI]	PRR [95% CI]	PRR [95% CI]	PRR [95% CI]
Age	1.047^∗∗∗^ [1.041,1.052]	1.069^∗∗∗^ [1.065,1.073]	1.079^∗∗∗^ [1.071,1.087]	1.049^∗∗∗^ [1.044,1.054]	1.016^∗∗∗^ [1.014,1.019]	1.028^∗∗∗^ [1.025,1.031]	0.989^∗∗∗^ [0.986,0.991]
Female (ref = male)	1.131^∗^ [1.018,1.257]	0.908^∗∗^ [0.851,0.970]	0.767^∗∗∗^ [0.673,0.874]	1.328^∗∗∗^ [1.190,1.483]	1.597^∗∗∗^ [1.466,1.739]	0.927^∗^ [0.868,0.990]	1.301^∗∗∗^ [1.217,1.390]
Not married or cohabiting (ref = married/cohabiting)	1.260^∗∗∗^ [1.129,1.405]	1.034 [0.963,1.111]	1.313^∗∗∗^ [1.149,1.501]	1.547^∗∗∗^ [1.362,1.758]	1.919^∗∗∗^ [1.770,2.081]	0.908^∗^ [0.836,0.987]	1.090^∗^ [1.013,1.173]
Non-tertiary education (ref = Tertiary education)	1.377^∗∗∗^ [1.171,1.619]	1.355^∗∗∗^ [1.231,1.493]	1.420^∗∗∗^ [1.159,1.741]	2.001^∗∗∗^ [1.641,2.440]	1.790^∗∗∗^ [1.598,2.006]	1.566^∗∗∗^ [1.440,1.703]	0.791^∗∗∗^ [0.729,0.857]
**Model 2**							
Age	1.044^∗∗∗^ [1.039,1.049]	1.067^∗∗∗^ [1.063,1.071]	1.074^∗∗∗^ [1.067,1.081]	1.045^∗∗∗^ [1.041,1.049]	1.017^∗∗∗^ [1.015,1.019]	1.028^∗∗∗^ [1.025,1.031]	0.989^∗∗∗^ [0.987,0.992]
Female (ref = male)	1.060 [0.957,1.175]	0.886^∗∗∗^ [0.828,0.948]	0.731^∗∗∗^ [0.640,0.834]	1.156^∗∗^ [1.036,1.289]	1.517^∗∗∗^ [1.393,1.652]	0.918^∗^ [0.860,0.981]	1.283^∗∗∗^ [1.202,1.371]
Not married or cohabiting (ref = married/cohabiting)	1.157^∗^ [1.035,1.293]	1.002 [0.932,1.078]	1.231^∗∗^ [1.078,1.406]	1.302^∗∗∗^ [1.138,1.490]	1.780^∗∗∗^ [1.644,1.928]	0.895^∗∗^ [0.823,0.973]	1.063 [0.985,1.147]
Non-tertiary education (ref = Tertiary education)	1.226^∗^ [1.044,1.439]	1.302^∗∗∗^ [1.181,1.436]	1.300^∗^ [1.064,1.588]	1.535^∗∗∗^ [1.262,1.868]	1.603^∗∗∗^ [1.431,1.796]	1.542^∗∗∗^ [1.419,1.674]	0.767^∗∗∗^ [0.707,0.832]
Workless (ref = employed)	1.793^∗∗∗^ [1.633,1.969]	1.272^∗∗∗^ [1.173,1.379]	1.664^∗∗∗^ [1.434,1.931]	3.554^∗∗∗^ [3.038,4.158]	1.643^∗∗∗^ [1.516,1.780]	1.092^∗^ [1.015,1.174]	1.152^∗∗∗^ [1.067,1.243]
**Model 3**							
Average employment rate	0.978^∗^ [0.959,0.997]	0.986 [0.971,1.002]	0.981 [0.955,1.008]	0.988 [0.972,1.004]	0.984^∗^ [0.970,0.999]	0.975^∗∗∗^ [0.965,0.984]	0.993 [0.977,1.010]
Age	1.044^∗∗∗^ [1.039,1.049]	1.067^∗∗∗^ [1.063,1.071]	1.074^∗∗∗^ [1.067,1.081]	1.045^∗∗∗^ [1.041,1.049]	1.017^∗∗∗^ [1.015,1.019]	1.028^∗∗∗^ [1.025,1.031]	0.989^∗∗∗^ [0.987,0.992]
Female (ref = male)	1.060 [0.957,1.175]	0.886^∗∗∗^ [0.828,0.948]	0.731^∗∗∗^ [0.641,0.835]	1.157^∗∗^ [1.037,1.290]	1.517^∗∗∗^ [1.393,1.652]	0.918^∗^ [0.860,0.980]	1.283^∗∗∗^ [1.202,1.370]
Not married or cohabiting (ref = married/cohabiting)	1.156^∗^ [1.034,1.292]	1.002 [0.931,1.078]	1.230^∗∗^ [1.077,1.405]	1.301^∗∗∗^ [1.137,1.489]	1.779^∗∗∗^ [1.642,1.926]	0.894^∗∗^ [0.822,0.972]	1.062 [0.984,1.146]
Non-tertiary education (ref = Tertiary education)	1.215^∗^ [1.036,1.425]	1.296^∗∗∗^ [1.175,1.430]	1.290^∗^ [1.055,1.577]	1.526^∗∗∗^ [1.255,1.855]	1.595^∗∗∗^ [1.424,1.787]	1.532^∗∗∗^ [1.411,1.663]	0.765^∗∗∗^ [0.705,0.831]
Workless (ref = employed)	1.782^∗∗∗^ [1.623,1.957]	1.268^∗∗∗^ [1.170,1.374]	1.655^∗∗∗^ [1.427,1.919]	3.539^∗∗∗^ [3.024,4.142]	1.636^∗∗∗^ [1.510,1.772]	1.087^∗^ [1.011,1.169]	1.150^∗∗∗^ [1.066,1.241]
**Model 4**							
Average employment rate	0.976^∗^ [0.957,0.995]	0.987 [0.971,1.003]	0.982 [0.954,1.011]	0.973^∗^ [0.952,0.994]	0.975^∗∗^ [0.959,0.991]	0.973^∗∗∗^ [0.962,0.984]	0.994 [0.977,1.011]
Age	1.044^∗∗∗^ [1.039,1.049]	1.067^∗∗∗^ [1.063,1.071]	1.074^∗∗∗^ [1.067,1.081]	1.045^∗∗∗^ [1.041,1.049]	1.017^∗∗∗^ [1.015,1.020]	1.028^∗∗∗^ [1.025,1.031]	0.989^∗∗∗^ [0.987,0.992]
Female (ref = male)	1.061 [0.958,1.175]	0.886^∗∗∗^ [0.828,0.948]	0.731^∗∗∗^ [0.640,0.835]	1.158^∗∗^ [1.038,1.291]	1.519^∗∗∗^ [1.395,1.653]	0.918^∗^ [0.860,0.980]	1.283^∗∗∗^ [1.201,1.370]
Not married or cohabiting (ref = married/cohabiting)	1.156^∗^ [1.034,1.292]	1.002 [0.931,1.078]	1.231^∗∗^ [1.078,1.405]	1.300^∗∗∗^ [1.136,1.488]	1.777^∗∗∗^ [1.640,1.924]	0.893^∗∗^ [0.821,0.972]	1.062 [0.984,1.146]
Non-tertiary education (ref = Tertiary education)	1.215^∗^ [1.036,1.424]	1.296^∗∗∗^ [1.175,1.431]	1.290^∗^ [1.056,1.577]	1.519^∗∗∗^ [1.251,1.845]	1.589^∗∗∗^ [1.420,1.779]	1.531^∗∗∗^ [1.411,1.661]	0.766^∗∗∗^ [0.705,0.831]
Workless (ref = employed)	1.401 [0.507,3.872]	1.384 [0.593,3.230]	1.876 [0.468,7.515]	0.839 [0.224,3.143]	0.512 [0.248,1.055]	0.816 [0.437,1.522]	1.212 [0.650,2.261]
Average employment rate * workless (ref = employed)	1.004 [0.989,1.019]	0.999 [0.986,1.011]	0.998 [0.977,1.019]	1.022^∗^ [1.002,1.042]	1.018^∗∗^ [1.006,1.029]	1.004 [0.995,1.014]	0.999 [0.990,1.009]
*N (individuals)*	22433	22433	22433	22793	22483	22169	22433
*N (regions)*	163	163	163	163	163	163	163

^∗^*p* < 0.05, ^∗∗^*p* < 0.01, ^∗∗∗^*p* < 0.001; CI=Confidence interval; PRR=Prevalence Risk Ratio; All models contain country fixed effects.

Worklessness was associated with higher risk of all adverse health outcomes; the strongest association was observed for poor self-rated health (3.554, 95% CI: 3.038 to 4.158) and the weakest for obesity (1.092, 95% CI: 1.015 to 1.174). When the average regional employment rate was added to the models the association between individual worklessness and the health outcomes decreased (Table 4, Model 3). Regional employment retained an association with heart and circulation problems (0.978, 95% CI: 0.959 to 0.997), depression (0.984, 95% CI: 0.970 to 0.999) and obesity (0.975^,^ 95% CI: 0.965 to 0.984), over and above the association with individual level worklessness. Sensitivity analysis breaking the workless group down demonstrated that the regional employment level remained associated with both heart and circulation problems and obesity (Table S2). The association with depression was weakened and no longer statistically significant at p < 0.05. This may be due to the strong association between depression and being out of work due to sickness, disability or unemployment.

**Fig. 2:**
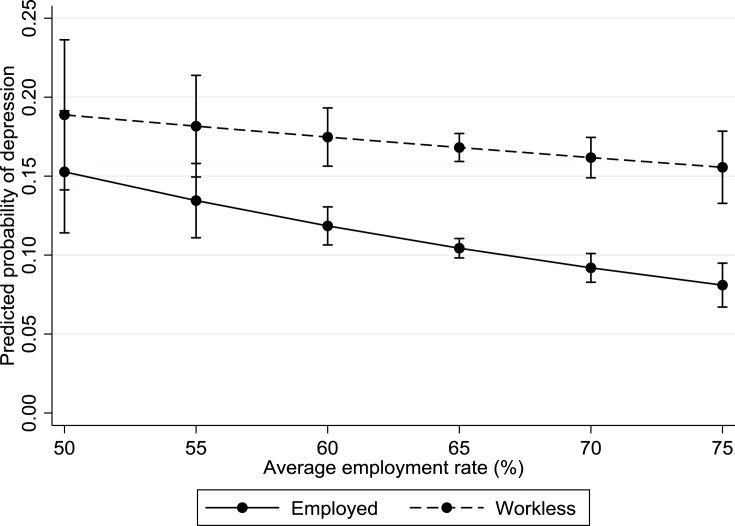
Predicted probability of depression for employed and workless groups according to the average regional employment rate from 2008 to 2013 (95% confidence intervals shown).

When we examined the interaction between individual level worklessness and the regional employment rate, we found statistically significant interactions for depression and poor self-rated health (Table 4, Model 4). For depression, at higher levels of regional employment the difference in depression and between the employed and workless was larger, with the employed experiencing lower risk of depression at higher levels of regional employment to a greater extent compared to the workless (Fig. 2). The sensitivity analysis breaking the workless group down revealed that no specific group was driving the interaction found for poor self-rated health. However, for depression, increased regional employment was related to higher risk of depression for the workless groups who were permanently sick or disabled or classified as ‘other’ (Table S3), which includes those in education. Whereas, the other groups experienced lower risk at higher rates of regional employment. In sensitivity analyses, we found no statistically significant interactions between gender, education level and regional employment levels (Table S4 and Table S5).

## 4 Discussion

Our analysis of worklessness, regional employment and health across 16 European countries revealed that higher regional employment levels were associated with lower risk of depression, heart and circulation problems and obesity, over and above the individual level association with worklessness, as well as country level factors. We also found that individual level worklessness was associated with each health outcome studied. Our results demonstrate that higher regional employment levels during 2008 and 2013, as well as the average regional employment rate across the period of the Great Recession, were associated with reduced risk of most health outcomes. The only outcome not associated with the average regional employment rate was allergies, which we included as a falsification test, and this therefore adds confidence to our findings. In addition, the change in regional employment did not seem to matter above that of the overall regional employment rate in the countries studied. When we investigated the interaction between the regional employment rate and individual level worklessness we found interesting results for the depression and poor self-rated health outcomes. For depression in particular, as regional employment levels increased the inequality in depression between the employed and workless increased, as the employed appeared to benefit more from the higher levels of regional employment. However, when we investigated this in further detail, the results seemed to be driven by the permanently sick or disabled group; the risk of depression increased as regional employment levels increased.

The finding that higher regional employment levels may be protective against poor health highlights the importance of considering potential contextual influences on health at different geographic levels. Although our study, to our knowledge, is the first cross-national study to examine the influence of regional employment on a range of health outcomes, the results are generally consistent with previous research limited to single countries. Research from England and Scotland demonstrated that neighbourhood unemployment was related to poor self-rated health, in addition to individual level unemployment (Cummins et al., 2005). Other studies find that higher local area unemployment may confer some protection against psychological distress associated with being unemployed (Flint et al., 2013). Similarly, the impact of unemployment on wellbeing was found to be less in areas of high unemployment in South Africa (Powdthavee, 2007). Evidence from Sweden demonstrated that higher vacancy rates at the municipal level was related to better mental health among the unemployed, but the unemployment rate had little influence (Strandh et al., 2011). Our findings for depression echo those of Buffel et al. (2016) who found that the employed were more depressed in regions with high unemployment rates, resulting in a narrowing of the mental health gap between the unemployed and the employed.

Taken together, our findings and those of other studies (as detailed above) highlight that the local labour market may be just as important for health as individual employment status (particularly for more sensitive outcomes such as mental health and those related to unhealthy behaviours). Further, people who are employed but living in households where another household member is unemployed or out of work due to other factors (e.g. illness or caring responsibilities) may also be impacted indirectly by declining regional employment levels. This could be due to the stress and worry related to the local labour market that may lead to increases in depression and anxiety, as well as the adoption of less healthy behaviours (e.g. poor diet) as a coping mechanism, and lower physical activity due to a decrease in work-related exertion (Colman and Dave, 2013), which could lead to obesity. Our findings are also supported by a recent study which used Scottish longitudinal data across the period of the Great Recession (Curtis et al., 2019). It found that for people living in areas that had experienced relatively high and stable levels of employment, the likelihood of reporting a mental illness was significantly lower when compared to similar people living in areas with persistently low employment rates. The authors also note that the trajectory of local area employment during the recession seems to have a stronger association with mental health compared to individual level employment status. Our study found that the change in regional employment during the time of the Great Recession did not have an additional association with the health outcomes under study, above that of the overall rate. However, the direction of the association between the change in regional employment and the more sensitive health outcomes in our study, including depression and obesity, was such that a rise in regional employment was suggestive of a protective association. These results reinforce the contextual effects literature which asserts that population health is shaped by both individual and area-level factors (Bambra, 2016).

The lack of a clear association for the change in employment rates in this study may be due to the very mixed impact of, and response to, the recession on different regions across Europe (Davies, 2011). The economic downturn associated with the Great Recession caused regional employment to drop in most countries across Europe (the average drop in employment between 2008 and 2013 was 1.7%), the highest regional decline occurred in Spain (−9.5%) and increased the most in Germany (3.6%). Macroeconomic policies which influence the demand for labour may be consequently important for health. Recessions impact population health unevenly depending on whether an economic stimulus approach was followed or austerity (Stuckler and Basu, 2013). Further work is needed to explore the unequal regional consequences of austerity for health and other potential mechanisms through which the regional labour market may influence health and health behaviours.

### 4.1 Strengths and limitations

The key strength of our study was the use of comparable cross-national data that integrated individual and regional level variables and included a range of health outcomes, whilst controlling for country fixed effects. We also investigated the full range of employment statuses, unlike previous studies which have often been limited to the employed and unemployed. Although we investigated a broad workless group, we also examined specific employment status groups to see if any were dictating the key results. Our study is unfortunately limited by the use of self-reported data which may be subject to reporting bias. However, our research builds on existing cross-national studies in the area of employment and health in which self-rated health has often been the sole outcome variable (Huijts et al., 2015; Shaw et al., 2014; Tøge and Blekesaune, 2015). Our study is also cross-sectional which precludes the inference of causal relationships. It also should be noted that the European Social Survey was never sampled for analysis at the regional level, so it may be possible that the respondents in some countries are not representative of the population at the sub-national level (Thomson et al., 2017). Data for some of the countries included was also only available at the largest NUTS level and we had to exclude a number of countries from the analysis due to a lack of comparable regional data. In addition, as we only examined regional employment levels we cannot rule out the possibility that our results are affected by other regional level factors, such as air pollution, Gross Domestic Product, income inequality and working conditions. However, it is likely that these may be causally related to regional employment levels and may therefore represent potential mediators rather than confounders. Similarly, although we included country fixed effects in our analyses to control for potential country level heterogeneity and to help reduce the possibility of omitted variable bias, there is still a possibility of residual confounding due to factors at the national level, such as the welfare and healthcare systems.

Our study is one of the first to investigate the impact of regional employment levels on a variety of health outcomes across the European working-age population. There was evidence to suggest that higher regional employment levels may exhibit a protective influence on some health outcomes, such as obesity and depression. These outcomes may be considered more sensitive to changes in the local environment, whereas the other outcomes such as diabetes may not be as sensitive with longer lag times if any effect is present. Future research would benefit from taking a life course approach to explore whether there are particular phases of the life course in which worklessness and regional labour markets relate to poor health (e.g. upon leaving education or late working life) (Pearce et al., 2018). Further research is needed to test potential causal links between regional employment and health by examining other factors in the local and regional environment which may be affected by employment levels, such as job insecurity, social cohesion, income and welfare spending (Milner et al., 2014; Niedzwiedz et al., 2016). More in-depth investigation of the potential biological mechanisms would also be fruitful (Hughes et al., 2017; Niedzwiedz et al., 2017). However, at present, there are a lack of comparable cross-national health data amongst the working-age population, with outcomes often limited to self-rated health. We also did not explore the impact of changing regional inequality in response to the recession. However, evidence suggests, at least for some Central and Eastern European countries, that regional variations in unemployment decreased following the Great Recession (Blažek and Netrdová, 2012). It will be important to fully explicate the long-term population health effects of the austerity measures implemented across some European countries and their likely uneven spatial distribution.

## 5 Conclusion

This cross-European study emphasises the importance of macroeconomic processes in understanding population health in Europe, but that there can be distinct geographical differences in the impact of these processes. In particular, our findings emphasise that worklessness is likely to be an important determinant of a variety physical and mental health outcomes. In addition, the work suggests that macroeconomic factors might exert an additional influence on the health of Europeans through the contextual influence of regional employment levels. This study therefore emphasises the importance of examining the geographical specificities of the political economy, and in particular, the multiple socio-spatial pathways through which structural factors exert an influence on population health and inequalities. Further work in this area will not only provide greater clarity as to the pathways linking macroeconomic change and health, but will also help to identify geographically-specific factors that can exacerbate or mitigate against these processes. These insights can provide policy makers with new insights into how to foster greater resilience to structural changes including financial shocks, such as economic recession.

## Declarations of interest

None.
