# Supplementary material for: Regional employment and individual worklessness during the Great Recession and the health of the working-age population: Cross-national analysis of 16 European countries
Source: Soc Sci Med. 2020 Dec;267:112377. doi: 10.1016/j.socscimed.2019.112377 (PMC7116502; doi:10.1016/j.socscimed.2019.112377)
Supplement: Multimedia component 1 [file mmc1.docx]

**Supplementary material**

Table S1: Descriptive statistics for each health condition by country

|  | **Heart / circulation problems** | | **High blood pressure** | | **Diabetes** | | **Poor self-rated health** | | **Depression** | | **Obesity** | | **Allergies** | |
| --- | --- | --- | --- | --- | --- | --- | --- | --- | --- | --- | --- | --- | --- | --- |
|  | No (N) | Yes (N) | No (N) | Yes (N) | No (N) | Yes (N) | No (N) | Yes (N) | No (N) | Yes (N) | No (N) | Yes (N) | No (N) | Yes (N) |
| Austria | 1,293 | 68 | 1,227 | 134 | 1,339 | 22 | 1,323 | 44 | 1,218 | 127 | 1,199 | 127 | 1,239 | 122 |
| Belgium | 1,315 | 82 | 1,226 | 171 | 1,362 | 35 | 1,348 | 52 | 1,250 | 146 | 1,224 | 171 | 1,187 | 210 |
| Czech Republic | 1,546 | 45 | 1,437 | 154 | 1,529 | 62 | 1,581 | 51 | 1,243 | 292 | 1,387 | 169 | 1,459 | 132 |
| Germany | 2,055 | 216 | 1,868 | 403 | 2,182 | 89 | 2,111 | 169 | 1,980 | 289 | 1,870 | 353 | 1,877 | 394 |
| Denmark | 1,102 | 51 | 1,021 | 132 | 1,108 | 45 | 1,098 | 57 | 1,027 | 121 | 999 | 135 | 933 | 220 |
| Spain | 1,404 | 69 | 1,332 | 141 | 1,433 | 40 | 1,393 | 90 | 1,223 | 230 | 1,240 | 208 | 1,295 | 178 |
| Finland | 1,414 | 88 | 1,262 | 240 | 1,431 | 71 | 1,464 | 38 | 1,396 | 98 | 1,211 | 261 | 1,204 | 298 |
| France | 1,346 | 90 | 1,302 | 134 | 1,387 | 49 | 1,353 | 84 | 1,255 | 177 | 1,223 | 181 | 1,206 | 230 |
| United Kingdom | 1,483 | 77 | 1,362 | 198 | 1,493 | 67 | 1,459 | 111 | 1,307 | 250 | 1,194 | 291 | 1,362 | 198 |
| Hungary | 1,137 | 80 | 1,032 | 185 | 1,170 | 47 | 1,161 | 81 | 1,002 | 219 | 1,055 | 158 | 1,143 | 74 |
| Ireland | 1,660 | 48 | 1,584 | 124 | 1,678 | 30 | 1,729 | 39 | 1,566 | 173 | 1,441 | 179 | 1,610 | 98 |
| Netherlands | 1,331 | 83 | 1,243 | 171 | 1,359 | 55 | 1,337 | 77 | 1,272 | 141 | 1,242 | 172 | 1,214 | 200 |
| Norway | 1,099 | 43 | 1,037 | 105 | 1,128 | 14 | 1,083 | 59 | 1,058 | 78 | 989 | 128 | 895 | 247 |
| Poland | 1,120 | 140 | 1,099 | 161 | 1,228 | 32 | 1,200 | 70 | 1,040 | 183 | 1,061 | 191 | 1,112 | 148 |
| Portugal | 600 | 62 | 544 | 118 | 622 | 40 | 782 | 54 | 648 | 187 | 703 | 123 | 521 | 141 |
| Sweden | 1,236 | 50 | 1,150 | 136 | 1,257 | 29 | 1,252 | 43 | 1,156 | 131 | 1,123 | 161 | 1,046 | 240 |
| Total | 21,141 | 1,292 | 19,726 | 2,707 | 21,706 | 727 | 21,674 | 1,119 | 19,641 | 2,842 | 19,161 | 3,008 | 19,303 | 3,130 |

Table S2: Results from multilevel regression models investigating individual level employment status, regional employment levels and health outcomes

|  | **Heart or circulation problems** | **High blood pressure** | **Diabetes** | **Poor self-rated health** | **Depression** | **Obesity** | **Allergies** |
| --- | --- | --- | --- | --- | --- | --- | --- |
|  | **PRR**  **[95% CI]** | **PRR**  **[95% CI]** | **PRR**  **[95% CI]** | **PRR**  **[95% CI]** | **PRR**  **[95% CI]** | **PRR**  **[95% CI]** | **PRR**  **[95% CI]** |
| Average employment rate | 0.980^*^ [0.961,0.999] | 0.988 [0.973,1.004] | 0.984 [0.958,1.010] | 0.999 [0.986,1.013] | 0.990 [0.976,1.004] | 0.977^***^ [0.967,0.986] | 0.993 [0.977,1.010] |
| Age | 1.039^***^ [1.033,1.045] | 1.066^***^ [1.061,1.070] | 1.069^***^ [1.061,1.078] | 1.029^***^ [1.022,1.036] | 1.008^***^ [1.005,1.012] | 1.022^***^ [1.019,1.026] | 0.987^***^ [0.984,0.991] |
| Female (ref=male) | 1.086 [0.979,1.205] | 0.897^**^ [0.836,0.962] | 0.730^***^ [0.635,0.840] | 1.265^***^ [1.143,1.401] | 1.565^***^ [1.441,1.699] | 0.912^**^ [0.853,0.974] | 1.286^***^ [1.204,1.372] |
| Not married or cohabiting (ref=married/cohabiting) | 1.132^*^ [1.015,1.262] | 0.989 [0.917,1.067] | 1.228^**^ [1.071,1.407] | 1.181^**^ [1.041,1.341] | 1.769^***^ [1.645,1.902] | 0.926 [0.854,1.005] | 1.061 [0.982,1.146] |
| Non-tertiary education (ref=Tertiary education) | 1.204^*^ [1.025,1.415] | 1.284^***^ [1.163,1.417] | 1.268^*^ [1.036,1.552] | 1.427^***^ [1.175,1.732] | 1.585^***^ [1.415,1.776] | 1.532^***^ [1.411,1.664] | 0.765^***^ [0.705,0.830] |
| Unemployed (ref=employed) | 1.616^***^ [1.325,1.969] | 1.285^***^ [1.129,1.463] | 1.391^*^ [1.056,1.832] | 2.641^***^ [2.151,3.243] | 2.004^***^ [1.814,2.214] | 1.198^**^ [1.062,1.351] | 1.090 [0.954,1.246] |
| Permanently sick or disabled (ref=employed) | 3.130^***^ [2.602,3.765] | 1.897^***^ [1.667,2.160] | 2.951^***^ [2.328,3.739] | 14.730^***^ [12.194,17.794] | 3.989^***^ [3.497,4.550] | 1.513^***^ [1.296,1.767] | 1.314^***^ [1.119,1.543] |
| Retired (ref=employed) | 1.888^***^ [1.620,2.199] | 1.199^***^ [1.079,1.332] | 1.640^***^ [1.315,2.045] | 2.975^***^ [2.351,3.766] | 1.445^***^ [1.250,1.670] | 1.181^**^ [1.048,1.329] | 1.332^***^ [1.136,1.562] |
| Homemaker (ref=employed) | 1.493^***^ [1.214,1.837] | 1.156^*^ [1.002,1.334] | 1.693^***^ [1.274,2.250] | 1.928^***^ [1.527,2.434] | 1.423^***^ [1.264,1.602] | 1.182^*^ [1.023,1.365] | 1.119 [0.987,1.270] |
| Other (ref=employed) | 1.342^*^ [1.033,1.745] | 1.007 [0.805,1.260] | 0.853 [0.536,1.360] | 1.349 [0.967,1.883] | 0.899 [0.762,1.060] | 0.587^***^ [0.470,0.734] | 1.082 [0.963,1.215] |
| *N (individuals)* | 22433 | 22433 | 22433 | 22793 | 22483 | 22169 | 22433 |
| *N (regions)* | 163 | 163 | 163 | 163 | 163 | 163 | 163 |

^*^ *p* < 0.05, ^**^ *p* < 0.01, ^***^ *p* < 0.001; PRR=Prevalence Risk Ratio; All models contain country fixed effects;

Table S3: Results from multilevel regression models investigating individual employment status and the interaction with regional employment for health

|  | **Poor self-rated health**  **PRR**  **[95% CI]** | **Depression**  **PRR**  **[95% CI]** |
| --- | --- | --- |
| Average employment rate | 0.988 [0.968,1.009] | 0.980^*^ [0.965,0.996] |
| Unemployed (ref=employed) | 0.665 [0.125,3.544] | 0.942 [0.366,2.422] |
| Permanently sick or disabled (ref=employed) | 4.592 [0.964,21.880] | 0.526 [0.180,1.538] |
| Retired (ref=employed) | 1.089 [0.146,8.096] | 1.449 [0.386,5.443] |
| Homemaker (ref=employed) | 1.329 [0.275,6.420] | 1.672 [0.529,5.288] |
| Other (ref=employed) | 0.922 [0.106,8.023] | 0.077^***^ [0.018,0.324] |
| Unemployed * Average employment rate (ref=employed) | 1.021 [0.996,1.047] | 1.012 [0.997,1.027] |
| Permanently sick or disabled * Average employment rate (ref=employed) | 1.017 [0.995,1.041] | 1.031^***^ [1.015,1.047] |
| Retired * Average employment rate (ref=employed) | 1.015 [0.985,1.046] | 1.000 [0.980,1.020] |
| Homemaker * Average employment rate (ref=employed) | 1.005 [0.982,1.030] | 0.997 [0.980,1.015] |
| Other * Average employment rate (ref=employed) | 1.006 [0.974,1.038] | 1.037^***^ [1.016,1.060] |
| Age | 1.029^***^ [1.022,1.037] | 1.008^***^ [1.005,1.012] |
| Female (ref=male) | 1.262^***^ [1.140,1.397] | 1.562^***^ [1.439,1.696] |
| Not married or cohabiting (ref=married/cohabiting) | 1.178^*^ [1.038,1.336] | 1.766^***^ [1.641,1.900] |
| Non-tertiary education (ref=Tertiary education) | 1.426^***^ [1.176,1.729] | 1.576^***^ [1.408,1.765] |
| *N (individuals)* | 22793 | 22483 |
| *N (regions)* | 163 | 163 |

^*^ *p* < 0.05, ^**^ *p* < 0.01, ^***^ *p* < 0.001; PRR=Prevalence Risk Ratio; All models contain country fixed effects;

Table S4: Results from multilevel regression models investigating gender and the interaction with regional employment for health

|  | **Heart or circulation problems** | **High blood pressure** | **Diabetes** | **Poor self-rated health** | **Depression** | **Obesity** | **Allergies** |
| --- | --- | --- | --- | --- | --- | --- | --- |
|  | **PRR**  **[95% CI]** | **PRR**  **[95% CI]** | **PRR**  **[95% CI]** | **PRR**  **[95% CI]** | **PRR**  **[95% CI]** | **PRR**  **[95% CI]** | **PRR**  **[95% CI]** |
| Average employment rate | 0.984 [0.964,1.004] | 0.991 [0.973,1.009] | 0.988 [0.961,1.016] | 0.984 [0.966,1.003] | 0.986 [0.970,1.003] | 0.973^***^ [0.962,0.983] | 0.997 [0.981,1.014] |
| Female (ref=male) | 2.202 [0.950,5.107] | 1.657 [0.866,3.169] | 2.086 [0.549,7.922] | 0.740 [0.257,2.131] | 1.871 [0.877,3.995] | 0.688 [0.380,1.245] | 2.033^*^ [1.081,3.826] |
| Female (ref=male)*average employment rate | 0.989 [0.976,1.002] | 0.991 [0.981,1.000] | 0.984 [0.965,1.005] | 1.007 [0.991,1.022] | 0.997 [0.986,1.008] | 1.004 [0.996,1.013] | 0.993 [0.984,1.003] |
| Age | 1.044^***^ [1.039,1.049] | 1.067^***^ [1.063,1.071] | 1.074^***^ [1.067,1.081] | 1.045^***^ [1.041,1.049] | 1.017^***^ [1.015,1.019] | 1.028^***^ [1.025,1.031] | 0.989^***^ [0.987,0.992] |
| Not married or cohabiting (ref=married/cohabiting) | 1.155^*^ [1.033,1.292] | 1.001 [0.931,1.077] | 1.230^**^ [1.077,1.405] | 1.302^***^ [1.138,1.490] | 1.778^***^ [1.642,1.926] | 0.894^**^ [0.822,0.972] | 1.062 [0.984,1.146] |
| Non-tertiary education (ref=Tertiary education) | 1.216^*^ [1.037,1.426] | 1.296^***^ [1.175,1.430] | 1.290^*^ [1.055,1.577] | 1.526^***^ [1.254,1.856] | 1.596^***^ [1.425,1.787] | 1.532^***^ [1.411,1.663] | 0.766^***^ [0.706,0.831] |
| Workless (ref=employed) | 1.781^***^ [1.621,1.956] | 1.266^***^ [1.168,1.372] | 1.652^***^ [1.424,1.916] | 3.540^***^ [3.025,4.142] | 1.636^***^ [1.510,1.772] | 1.087^*^ [1.011,1.169] | 1.150^***^ [1.065,1.241] |
| *N (regions)* | 163 | 163 | 163 | 163 | 163 | 163 | 163 |
| *N (individuals)* | 22433 | 22433 | 22433 | 22793 | 22483 | 22169 | 22433 |

^*^ *p* < 0.05, ^**^ *p* < 0.01, ^***^ *p* < 0.001; PRR=Prevalence Risk Ratio; All models contain country fixed effects;

Table S5: Results from multilevel regression models investigating education level and the interaction with regional employment for health

|  | **Heart or circulation problems** | **High blood pressure** | **Diabetes** | **Poor self-rated health** | **Depression** | **Obesity** | **Allergies** |
| --- | --- | --- | --- | --- | --- | --- | --- |
|  | **PRR**  **[95% CI]** | **PRR**  **[95% CI]** | **PRR**  **[95% CI]** | **PRR**  **[95% CI]** | **PRR**  **[95% CI]** | **PRR**  **[95% CI]** | **PRR**  **[95% CI]** |
| Average employment rate | 0.968^**^ [0.945,0.991] | 0.984 [0.966,1.003] | 0.961^*^ [0.924,1.000] | 0.994 [0.961,1.028] | 0.991 [0.973,1.009] | 0.967^***^ [0.953,0.981] | 0.991 [0.973,1.010] |
| Non-tertiary education (ref=Tertiary education) | 0.543 [0.120,2.451] | 1.087 [0.413,2.860] | 0.247 [0.026,2.317] | 2.454 [0.283,21.315] | 2.627 [0.920,7.498] | 0.796 [0.373,1.696] | 0.640 [0.297,1.380] |
| Non-tertiary education (ref=Tertiary education) * average employment rate | 1.012 [0.990,1.035] | 1.003 [0.989,1.017] | 1.025 [0.992,1.059] | 0.993 [0.962,1.025] | 0.993 [0.977,1.009] | 1.010 [0.999,1.021] | 1.003 [0.991,1.014] |
| Female (ref=male) | 1.060 [0.957,1.174] | 0.886^***^ [0.828,0.948] | 0.731^***^ [0.641,0.835] | 1.157^**^ [1.038,1.290] | 1.518^***^ [1.394,1.653] | 0.918^**^ [0.859,0.979] | 1.283^***^ [1.202,1.370] |
| Age | 1.044^***^ [1.039,1.049] | 1.067^***^ [1.063,1.071] | 1.074^***^ [1.067,1.081] | 1.045^***^ [1.041,1.049] | 1.017^***^ [1.015,1.019] | 1.028^***^ [1.025,1.031] | 0.989^***^ [0.987,0.992] |
| Not married or cohabiting (ref=married/cohabiting) | 1.155^*^ [1.033,1.291] | 1.001 [0.931,1.077] | 1.229^**^ [1.076,1.404] | 1.302^***^ [1.138,1.490] | 1.780^***^ [1.643,1.928] | 0.893^**^ [0.821,0.971] | 1.062 [0.984,1.145] |
| Workless (ref=employed) | 1.784^***^ [1.625,1.959] | 1.268^***^ [1.170,1.374] | 1.657^***^ [1.430,1.921] | 3.539^***^ [3.023,4.143] | 1.635^***^ [1.509,1.772] | 1.088^*^ [1.012,1.170] | 1.151^***^ [1.066,1.242] |
| *N (regions)* | 163 | 163 | 163 | 163 | 163 | 163 | 163 |
| *N (individuals)* | 22433 | 22433 | 22433 | 22793 | 22483 | 22169 | 22433 |

^*^ *p* < 0.05, ^**^ *p* < 0.01, ^***^ *p* < 0.001; PRR=Prevalence Risk Ratio; All models contain country fixed effects
